# Inhibition or Stimulation of Autophagy Affects Early Formation of Lipofuscin-Like Autofluorescence in the Retinal Pigment Epithelium Cell

**DOI:** 10.3390/ijms18040728

**Published:** 2017-03-29

**Authors:** Lei Lei, Radouil Tzekov, Huapeng Li, J. Hugh McDowell, Guangping Gao, W. Clay Smith, Shibo Tang, Shalesh Kaushal

**Affiliations:** 1 State Key Laboratory of Ophthalmology, Zhongshan Ophthalmic Center, Sun Yat-sen University, No.54 South Xianlie Road, Guangzhou 510060, China; leilei@gzzoc.com; 2 Department of Ophthalmology, University of Massachusetts Medical School, 381 Plantation Street, Worcester, MA 01605, USA; rtzekov@health.usf.edu; 3 Department of Ophthalmology, University of South Florida, 13127 USF Magnolia Drive, Tampa, FL 33612, USA; 4 The Roskamp Institute, 2040 Whitfield Avenue, Sarasota, FL 34243, USA; 5 Gene Therapy Center, University of Massachusetts Medical School, 381 Plantation Street, Worcester, MA 01605, USA; Huapeng.Li@umassmed.edu (H.L.); Guangping.Gao@umassmed.edu (G.G.); 6 The Department of Ophthalmology, University of Florida Health Science Center, 1600 SW Archer Road, Gainesville, FL 32610, USA; jmcdowel@ufl.edu (J.H.M.); wcsmith@ufl.edu (W.C.S.); 7 Aier School of Ophthalmology, Central South University, Floor 4, New Century Building, 198# Furong Middle Road, Changsha 410015, China; tangshibo@vip.163.com; 8 VRMI, 6205 NW 81st Drive, Gainesville, FL 32653, USA

**Keywords:** retinal pigment epithelium, lipofuscin, autofluorescence, age-related macular degeneration, mTOR, autophagy, degradation

## Abstract

The accumulation of lipofuscin in the retinal pigment epithelium (RPE) is dependent on the effectiveness of photoreceptor outer segment material degradation. This study explored the role of autophagy in the fate of RPE lipofuscin degradation. After seven days of feeding with either native or modified rod outer segments, ARPE-19 cells were treated with enhancers or inhibitors of autophagy and the autofluorescence was detected by fluorescence-activated cell sorting. Supplementation with different types of rod outer segments increased lipofuscin-like autofluorescence (LLAF) after the inhibition of autophagy, while the induction of autophagy (e.g., application of rapamycin) decreased LLAF. The effects of autophagy induction were further confirmed by Western blotting, which showed the conversion of LC3-I to LC3-II, and by immunofluorescence microscopy, which detected the lysosomal activity of the autophagy inducers. We also monitored LLAF after the application of several autophagy inhibitors by RNA-interference and confocal microscopy. The results showed that, in general, the inhibition of the autophagy-related proteins resulted in an increase in LLAF when cells were fed with rod outer segments, which further confirms the effect of autophagy in the fate of RPE lipofuscin degradation. These results emphasize the complex role of autophagy in modulating RPE autofluorescence and confirm the possibility of the pharmacological clearance of RPE lipofuscin by small molecules.

## 1. Introduction

The accumulation of lipofuscin in the retinal pigment epithelium (RPE) is associated with the normal process of aging, but can also be an element in the pathological process associated with several retinal degenerative diseases [1,2,3]. There is also evidence that lipofuscin accumulation can lead to RPE and photoreceptor cell death [4,5,6]. Many aspects of this important biological process are not well understood [5]. In particular, little is known about the cellular mechanisms that control the effective degradation of significant amounts of photoreceptor outer segment material by RPE during the process of photoreceptor outer segment renewal. In most mammals, each RPE cell opposes many dozens of rod photoreceptors and must process thousands of outer segment discs every day [7]. For example, in the primate perifovea, each RPE cell services about 40 rod cells [8] and the volume of the processed outer segment material on a daily basis (~10% of the length of each outer segment, phagocytized usually in a matter of few hours), which can be estimated to be equal to about 20% of the volume of one RPE cell. However, relatively little lipofuscin material accumulates in the RPE of normal subjects, especially during the first decade of life [9]. Therefore, despite the heavy metabolic overload imposed by the considerable volume of rod outer segment (ROS) material to be phagocytized, degraded, and recycled, a very efficient and tightly regulated mechanism exists to keep the level of undigested material very low, which is an essential condition for the proper functioning of an RPE cell.

However, some accumulation of lipofuscin material still occurs with normal aging and even more is seen in some pathological conditions, like age-related macular degeneration (AMD) [10,11], Stargardt’s disease [12,13], and even retinitis pigmentosa [14,15]. Therefore, it is likely that even small inefficiencies in the process of ROS degradation can compound over time and interfere with the functioning of the RPE cell layer and the overlying retina. The realization of this RPE vulnerability has stimulated an intensive research effort to understand the mechanisms which control the relatively efficient degradation under normal conditions and the pathological processes associated with their defects.

It has long been accepted that during the normal functioning of macrophages, different components of phagocytized material (lipids, amino acids, and carbohydrates) accumulate in the lysosomes [16,17]. Similarly, for a long time, the RPE lysosomes have been suspected to play a central role in the degradation of photoreceptor outer segment material [18]. The lysosomal complex participates in autophagy and there is significant indirect evidence to suggest that this process may play a central role in maintaining relatively low lipofuscin levels in normal RPE cells [19,20,21]. Theoretically, if the process of autophagy is suppressed, a gradual accumulation of lipofuscin material occurs over time. Conversely, if stimulation of this process occurs, the results include an increase in the speed and efficiency of the degradation of newly-formed lipofuscin, less lipofuscin accumulation in the RPE cells over time, and possibly some degradation of existing undegraded material.

Since the degradation of RPE lipofuscin is a complicated, multi-step process and autophagy is the most important intracellular catabolic process involved in protein and organelle degradation via the lysosomal pathway, manipulating the process of autophagy in RPE cells is being explored as a potential way to regulate early lipofuscin formation [22,23,24]. However, there is still a lack of direct evidence on whether inhibiting or stimulating autophagy increases or reduces the amount of lipofuscin-like material in the RPE cell. The goal of this work was to investigate some important aspects of this process. By adding different pharmacologic agents and conducting various experiments, we investigated several aspects of the role of lysosomes, autophagy, and the proteasome system in the fate of RPE lipofuscin degradation. Thus, this work could be a step forward towards defining new targets for drug development in AMD and other lipofuscin-related diseases.

## 2. Results

### 2.1. Effects of Autophagy Induction or Inhibition on RPE Cells Autofluorescence

**Figure 1:**
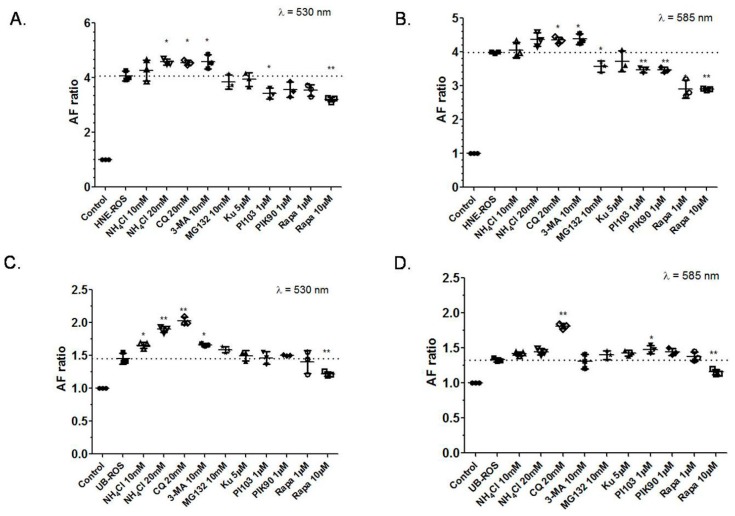
Effects of autophagy induction or inhibition on RPE cells autofluorescence detected by FACS. (**A**–**D**) Bar graphs of relative LLAF, expressed as a ratio of the fluorescence from cells supplemented with rod outer segments (ROS) and treated with small molecules relative to the fluorescence detected from cells treated with small molecules only. The fluorescence levels detected from cells supplemented with ROS only are presented with a horizontal dotted line. (**A**) After seven days of feeding with ROS modified with the lipid peroxidation product 4-hydroxynonenal (HNE), ARPE-19 cells were treated with ammonium chloride (NH_4_Cl, a lysosomal inhibitor), chloroquine (CQ, a lysosomal inhibitor), 3-MA (an autophagy inhibitor), MG-132(an autophagy inducer), Ku-0063794 (Ku, a mTOR kinase inhibitor), PI-103 (a dual phosphoinositide 3-kinase and mTOR inhibitor), PIK90 (a PI3Kα inhibitor), and rapamycin (Rapa, a known mTOR inhibitor and autophagy inducer) at doses indicated in the labels below the horizontal axis for 48 h and AF was measured by FACS at 533 nm; (**B**) Same conditions as in **A**, but detected at 585 nm; (**C**) Same treatment and detection as in **A**, but seven days feeding was with native ROS; (**D**) Same treatment and detection as in **B**, but feeding was with native ROS. Values are presented as means and standard deviations, based on three replicates. Significant differences in LLAF between different preparations and relative LLAF for cells fed with HNE-modified ROS in **A** and **B**, and with native ROS in **C** and **D**, are denoted with an asterisk/s (*post-hoc* Dunnett’s multiple comparisons test; * *p* < 0.05; ** *p* < 0.01).

To determine the intracellular fate of lipofuscin within the RPE cell, we investigated the correlation between the inhibition or stimulation of the autophagy and/or the proteasome system, on one hand, and the change in RPE autofluorescence on the other hand. To clarify the significance of this correlation, we used two different types of ROS for the RPE cells. First, in order to understand the role of autophagy modulation under more physiological conditions, we supplemented the RPE cells with native (unbleached) ROS for seven days and then applied various treatments related to the degradation. Second, to explore the possibility for a pharmacological modulation of RPE cells that have already accumulated undegraded products, mostly of ROS origin, we supplemented the cells with ROS modified with a lipid peroxidation product for seven days, namely 4-hydroxynonenal (HNE), a substance that can accumulate in the RPE or in some other diseases, such as neuronal ceroid lipofuscinosis [25]. The supplementation of HNE-modified ROS increased the LLAF from RPE cells to a much larger extent (~10 times) compared to the supplementation with native ROS, at both 530 and 585 nm (Figure 1), a result which is in line with the findings from similar experiments previously reported [26]. The application of the proteasomal inhibitor and autophagy inducer MG-132 [27] had a minimal effect on LLAF in cells supplemented with either HNE-modified ROS or native ROS (Figure 1A,B). In contrast, treatment with lysosomal inhibitors NH_4_Cl [28] and chloroquine [29], as well as with the autophagy inhibitor 3-MA [30], all increased LLAF to a varying degree under both supplementation paradigms (native ROS and HNE-modified ROS) and at both wavelengths, with chloroquine leading to the greatest LLAF increase when the RPE cells were supplemented with native ROS and approximately the same effect in cells supplemented with HNE-modified ROS (Figure 1).

Within the scope of the same experimental paradigm, we also treated the cells with autophagy inducers. A single application of rapamycin (10 µM), a known mTOR inhibitor and autophagy inducer [31,32], significantly decreased LLAF by 20–25% in cells fed with either native or HNE-modified ROS (Figure 1). However, three other compounds, all of which have been proven to be autophagy inducers in other cell systems: Ku-0063794, an mTOR kinase inhibitor [33]; PI-103, a dual phosphoinositide 3-kinase (PI3K) and mTOR inhibitor [34]; and PIK-90, a PI3Kα inhibitor with very low mTOR inhibitory activity [35], decreased the LLAF differently and slightly in cells supplemented with HNE-modified ROS (Figure 1A,B)or native ROS (Figure 1C,D). PI-103 decreased the LLAF with less potency compared to rapamycin, while PIK90 and Ku-0063794 didn’t exhibit much of an effect. Among all of the groups, rapamycin at 10 µM showed the strongest decrease in LLAF, indicating that mTOR inhibitors may play an important role in the degradation of lipofuscin. However, the specific pathway and mechanism need to be explored.

### 2.2. Effect of Rapamycin Treatment on RPE Autofluorescence by Live Cell Imaging

**Figure 2:**
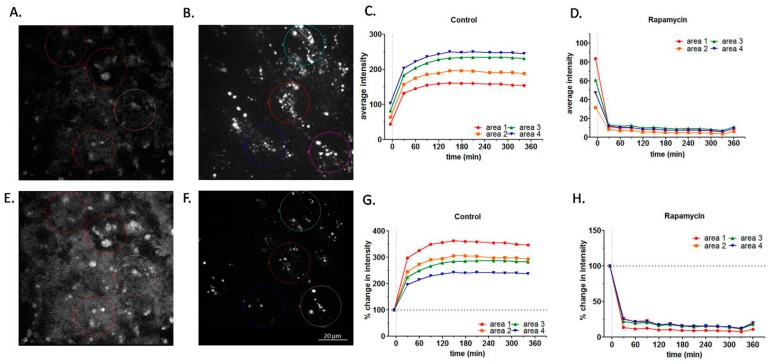
Effect of rapamycin treatment on RPE autofluorescence by live cell imaging. (**A**,**B**,**E**,**F**) Microphotographs of RPE autofluorescence obtained with live cell imaging at 610 nm before and after the addition of rapamycin or PBS. (**A**) Combined confocal control image before the addition of PBS; (**B**) Combined confocal control image before the addition of rapamycin (10 µM); (**E**) Combined confocal image at 342 min after the addition of PBS; (**F**) Combined confocal image at 360 min after the addition of rapamycin. Four color circles indicate the areas chosen for the quantitation of autofluorescence over time. (**C**,**D**,**G**,**H**) Quantification of autofluorescence in live cell imaging. Quantification of the RPE autofluorescence registered by live cell imaging presented on panels **A**,**B**,**E**,**F** and Supplemental Movies 1 and 2; (**C**) Changes in absolute intensity vs. time with PBS treatment (control) for each of the four colored circular regions outlined in panels **A**,**B**,**E**,**F**. Please note that the initial conditions in Panels **C** and **D** (time 0) are very similar; (**D**) Changes in absolute intensity vs. time with rapamycin treatment for the four circular regions; (**G**) Changes in relative intensity (post-treatment intensity for each circular region normalized towards the corresponding intensity pre-treatment) vs. time with PBS treatment; (**H**) Changes in relative intensity vs. time with rapamycin treatment (normalization as in **C**). Scale bar—20 µm. For more details, see the Methods section of the main text.

**Figure 3:**
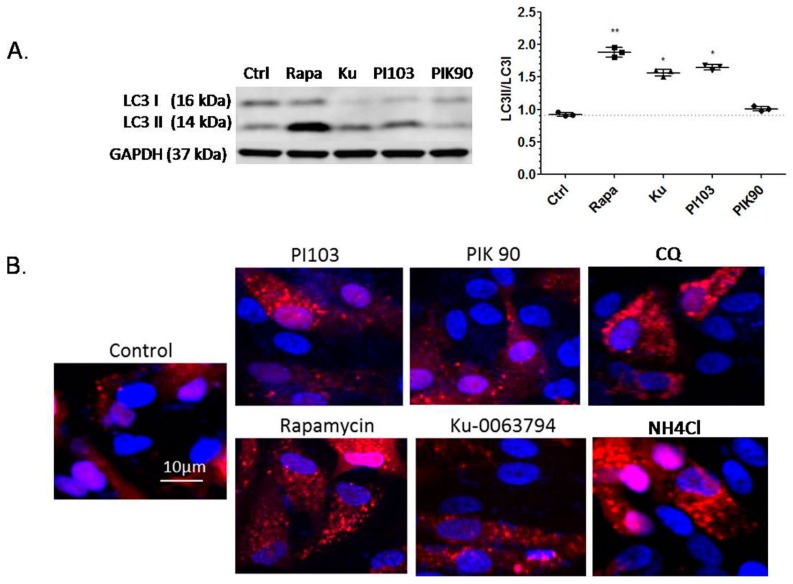
Effects of autophagy induction and lysosomal inhibitors on protein expression and lysosomal activity by confocal microscopy.(**A**) Western blot results for the microtubule-associated protein 1 light chain 3 (LC3). Upper part: LC3-1 (LC3-I) form (cytoplasmic form); lower part: LC3-2 (LC3-II) form (autophagosomal form) of the ARPE-19 cells at 8 h after no treatment (Ctrl), or after treatment with rapamycin (RAPA, 10 µM), KU-0063794 (Ku, 5 µM), PI-103 (PI103, 1 µM) and PIK-90 (1 µM); LC3-II/LC3-I ratios were calculated and the values are presented as means and standard deviations, based on three replicates; Significant differences between different groups are denoted with an asterisk/s (post-hoc Dunnett’s multiple comparisons test; * *p* < 0.05; ** *p* < 0.01); (**B**) Confocal microphotographs of ARPE-19 cells transfected with mCherry-LC3 at 48 h after the single addition of: 10 μM rapamycin, 1 µM PI-103, 1 µM PIK-90, 5 µM Ku-0063794, 10 µM of NH_4_Cl, 20 µM of chloroquine, and PBS (control). Note the punctate appearance of the fluorescence in cells treated with the drugs compared to the cells treated with PBS (control). Red color represents mCherry-LC3, blue indicates cell nuclei (DAPI).

To further investigate the role of rapamycin on RPE autofluorescence, live cell imaging for the untreated ARPE-19 cells was examined. It clearly demonstrated a rapid substantial decrease in LLAF as quickly as 30 min after the administration of rapamycin compared to the administration of PBS (Figure 2, Movie S1, Movie S2). Most of the decrease took place within the first 30 min after administration, indicating a rapid and efficient autophagy response (Figure 3), consistent with a short half-life (~10 min) of autophagosomes [36]. The difference in the degree of decrease in autofluorescence after the application of rapamycin in live cell imaging and the decrease detected by FACS in some of the experiments described above (Figure 1), can be attributed to several important physical factors varying between the two experimental conditions, especially the difference in the spectral emission and absorbance profiles of the filter systems. Furthermore, the initial increase in LLAF (first 120 min), as shown in Figure 2C,G, could be due to a combination of several factors: (a) exposure to the laser beam illumination as part of the focusing process when the live cell imaging slide is placed on the microscope stage; (b) the increase in the live tissue temperature (from room temperature to 37 °C) due to the action of the heater on the stage; (c) the application of PBS itself, which may have slightly agitated the cells and led to improved conditions for oxidation and, therefore, to increased autofluorescence. Future control experiments will be conducted to minimize the influence of these factors.

### 2.3. Effects of Autophagy Induction on Protein Expression and Lysosomal Activity by Confocal Microscopy

Western blot results of the conversion of lysosomal marker microtubule-associated protein light chain 3 (LC3), from its cytoplasmic LC3-I form to the autophagosomal LC3-II form, confirmed the induction of autophagy by rapamycin and by Ku-0063794 and PI-103 in the ARPE-19 cells (Figure 3A). To further explore the role of autophagy and lysosomes in the degradation of lipofuscin, a lentivirus-mCherry-LC3 plasmid was constructed and transfected into the ARPE-19 cells. When treated with either rapamycin, Ku-0063794, or PI-103, the ARPE-19 cells exhibited a punctate appearance (Figure 3B), which provided additional confirmation of the conversion of LC3. In the meanwhile, adding the lysosomal inhibitors NH_4_Cl and chloroquine to the cells also resulted in a diffuse punctate appearance of LC3 inside the cells, indicating lysosomal dilatation, as described in many cells, including RPE by Yoon et al. [37] (Figure 3B).

### 2.4. Effects of Autophagy Inhibition by RNA Interference on Autofluorescence of RPE Cells

**Figure 4:**
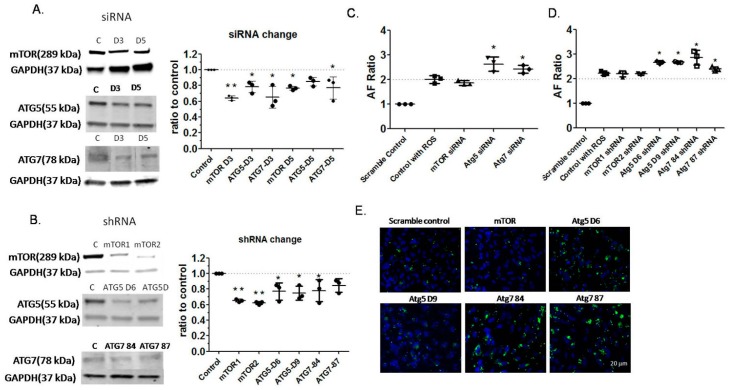
Autofluorescence of RPE cells transfected with siRNA and shRNA.(**A**) Western blot results demonstrating protein levels of mTOR, autophagy protein 5 (Atg5), and autophagy protein 7 (Atg7) at three days (D3) and five days (D5) after transfection with siRNA; (**B**) Western blot results demonstrating protein levels of mTOR (mTOR1, mTOR2), Atg5 for both lentiviral constructs (D6 and D9), and Atg7 for both lentiviral constructs (84 and 87) at three days after transfection with shRNA. Results presented in **A** and **B** showed that protein levels were reduced after transfection and the values are presented as means and standard deviations, based on three replicates. Significant differences between different groups are denoted with an asterisk/s (*post-hoc* Dunnett’s multiple comparisons test; * *p* < 0.05, ** *p* < 0.01); (**C**) Autofluorescence of ARPE-19 cells after three days of transfection with siRNA against mTOR, Atg5, and Atg7, and then three days feeding with HNE-modified ROS; three replicates; (**D**) Autofluorescence of ARPE-19 cells after three days of transfection with shRNA against mTOR, Atg5, and Atg7, and then three days feeding with HNE-modified ROS; three replicates. Results presented in **C** and **D** were obtained by FACS analysis in PE channel with an emission of 585/40 nm, and represent means and standard deviations. Significant differences in LLAF between different preparations and relative LLAF for cells fed scramble control in **C** and **D** (and for the **right panels** in **A** and **B**)are denoted with an asterisk (post-hoc Dunnett’s multiple comparisons test, * *p* < 0.05, ** *p* < 0.01). Analysis of the autofluorescence at 530 nm showed similar pattern as at 585 nm (data not shown); (**E**) Microphotographs of ARPE-19 cells at three days after transfection with shRNA and feeding for three consecutive days with HNE-ROS. LLAF (green color, detected by fluorescein filter set, see Methods) of cells (nuclei stained with DAPI—blue color) infected with Atg5 and Atg7 shRNA increased with little change in the growth of the cells, while LLAF of cells infected with mTOR shRNA decreased with a decrease in the growth of the cells because of the associated physiological effect. Scale bar—20 µm.

Based on the previous results showing an important role of rapamycin and autophagy, we then explored the effects of blocking the synthesis of mTOR and two autophagy-associated proteins: autophagy protein 5 (Atg5) [38] and autophagy protein 7 (Atg7) [39], on ARPE-19 cell LLAF by transfection with small interfering RNA (siRNA) or small hairpin RNA (shRNA). The siRNA short-term inhibition experiments demonstrated a 20–60% knockdown in the levels of mTOR, Atg5, or Atg7, as measured by Western blot tests (Figure 4A, Figures S1–S5). The knockdown based on shRNA transfection was similar (Figure 4B, Figures S1–S5). The LLAF of cells infected with either siRNA or shRNA against Atg5 or Atg7 increased with little change in the growth of the cells, while the LLAF of cells infected with siRNA or shRNA against mTOR decreased with a decrease in the growth of the cells because of the associated physiological effect (Figure 4C–E, Figure S6). In both cases, RPE cells showed increased LLAF values after the knockdown of Atg5 and Atg7, and a tendency for decreased LLAF values by the knockdown of mTOR, although this decrease was not statistically significant. These data provide further evidence that suppressing autophagy leads to the accumulation of lipofuscin-like material in the RPE cells.

## 3. Discussion

RPE lipofuscin is clinically recognized as an important factor in the pathogenesis of several retinal diseases, such as Stargardt’s disease, cone-rod dystrophy, and AMD [4,11,40]. Mounting clinical evidence shows that increased lipofuscin-related autofluorescence in human patients with AMD is a precursor for the eventual death of RPE and photoreceptor cells, which subsequently leads to central vision loss [41,42]. Similarly, parafoveal rings of high density autofluorescence are believed to occur in different retinal dystrophies and are likely related to their pathogenesis and progression [14,43]. However, the events leading to lipofuscin accumulation and clearance in RPE cells are not well characterized. The experiments conducted in this work were designed to address the issue in the context of a controlled cell culture environment. The results indicate that the inhibition of the process of autophagy by small molecules or by RNA interference leads to an increase in lipofuscin-like autofluorescence in RPE cells, in parallel with the accumulation of undegraded material in the lysosomes of the RPE cell. On the other hand, the stimulation of autophagy leads to a moderate decrease in LLAF, as shown by fluorescence-activated cell sorting (FACS) and cell imaging with confocal microscopy.

The proteasome system (sometimes referred to as the ubiquitin-proteasome system) is a cellular pathway responsible for degrading the majority of the short-lived cells and abnormal proteins, and operates through the 26S proteasome [44]. In contrast, the autophagy system is mostly responsible for the degradation of long-lived proteins and cellular organelles, and operates through lysosomes [45]. Both systems are present and operate in the RPE cells. For more details, the reader is directed to some recent reviews [23,46].

### 3.1. Inhibition of the Lysosomal Complex and Autophagy Increases LLAF

The application of the lysosome inhibitor chloroquine increased LLAF in cells fed with either native or lipid-oxidized ROS. In support of this observation, it has recently been demonstrated that the application of chloroquine to ARPE-19 cells in similar concentrations to the ones applied in the current study leads to an increase in vacuolation and dense intracellular debris identified as chloroquine-dilated lysosomes and lipid bodies [47]. Furthermore, it is well established that the systemic administration of chloroquine, which is mainly used as an anti-rheumatic agent, is associated with increased autofluorescence in humans and various alterations of central visual function [48].

We observed a similar increase in LLAF after the application of ammonium chloride, a known lysosomal inhibitor [28]. This result is in accordance with previous reports indicating the capability of ammonium chloride supplementation to induce lipofuscin-like material in RPE cells [25,49]. Finally, the application of 3-MA, an autophagy inhibitor, also resulted in an increase in LLAF comparable with the increase observed with both of the lysosomal inhibitors described above. This is in keeping with previous reports indicating that the administration of 3-MA inhibits the lysosomal function in RPE cells and leads to the accumulation of lipofuscin-like material [25,49].

Typically, when studying the accumulation of lipofuscin in RPE cells by FACS, the investigators report emissions from one wavelength (530 nm) [50]. In this study, we opted to investigate the autofluorescence at two wavelengths (530 and 585 nm),because the characteristic pattern of fluorescence detected from the human lipofuscin granules [25,51] or in patients [52], exhibits a broad peak around 580–620 nm, and, therefore, the inclusion of 585 nm would provide a better correlation with the fate of human lipofuscin in vivo. Furthermore, a recent study established an autofluorescence peak of 568 nm in AMD eyes and 572 nm for control eyes, values much closer to a wavelength of 585 nm than to 530 nm [53]. Generally, there appeared to be some similarity in fluorescence increases across both wavelengths. However, some differences were also observed. For example, the application of 3-MA resulted in a significant increase in LLAF after the supplementation with native ROS at 530 nm, but not at 585 nm. This underscores the complexity of the RPE lipofuscin formation process and the importance of evaluating multiple wavelengths to better understand and more completely evaluate the accruing changes.

### 3.2. Inhibition of mTOR and PIK3 α Pathways Decreases LLAF

We applied several inhibitors of the mTOR pathway to further explore the possibility of reduction in newly-formed lipofuscin-like material in RPE cells. The four inhibitors applied have a different potency and specificity against mTOR and its two major components, mTORC1 and mTORC2. The inhibitors can be ranked in order of potency (from strongest to weakest), as follows: rapamycin, PI-103, Ku-0063794, and PIK-90. Among these drugs, rapamycin at 10 µM consistently showed the most pronounced and significant decrease in LLAF, at both 535 and 585 nm, with both ROS supplementations (Figure 1).The effect was also confirmed directly by live cell imaging, pointing to a strong effect on autophagy induction. Of note, a lower dose of rapamycin (1 µM) did not demonstrate any effect on LLAF when applied to RPE cells treated with native ROS, but decreased LLAF at 585 nm in ROS cells supplemented with HNE-modified ROS (Figure 1). Recently, a rapamycin’s effect on ARPE-19 cells has been demonstrated, showing that it stimulates autophagy and can reduce A2E accumulation, and that rapamycin restored cell viability in RPE cells incubated with A2E [54]. In our work, similar effects were observed upon the application of PI103, which displayed a smaller decrease than rapamycin. However, the effect of Ku-0063794 and PIK-90 were not as obvious and stable as rapamycin. The similarity and difference of the effect between the four drugs is slightly surprising, given the difference in potency on the mTOR pathway, and is a possible indication that these drugs may act via slightly different mechanisms. The main difference between Ku-0063794 and rapamycin is the concentration required to suppress mTORC1 and mTORC2. While Ku-0063794 is equally effective in suppressing both components, rapamycin is much more potent against mTORC1. The same is true for PI-103, while PIK-90 does not directly inhibit mTOR at the dose used in this study. Therefore, it is likely that a difference between the activity of these mTOR inhibitors exists in RPE cells, in contrast to glioma cells, where it was demonstrated that they all induce autophagy to a similar extent [55].

Our observation that a similar decrease in LLAF is achieved by the application of equimolar amounts of rapamycin and other mTOR C1-independent inducers, such as PI-103, suggests that autophagy regulation in the RPE cell is controlled at multiple levels. Of particular interest is the reduction of LLAF with PI3Kα inhibitors. Some authors describe PI3Kα as a separate pathway that controls autophagy [56], although little is known about its function in the RPE cell. Our observation suggests that, in the RPE cells, PI3Kα and mTOR pathways are connected and likely interdependent.

The decrease in LLAF by rapamycin in cells fed with HNE-modified ROS both shortly after a single treatment (Figure 2) and after seven-day dosing (Figure 1A–D), is the first direct evidence that autophagy induction leads to the reduction of lipofuscin-like material in the RPE cell.

In the present study, the small molecule approach was complemented by siRNA and shRNA knockdown studies of Atg5 and Atg7, which are both key components of autophagy regulation [38,39]. In particular, Atg5 has been found in large drusen and in the sub-RPE space in humans [57], implicating it as a component involved in lipofuscin generation, and our results are consistent with this observation. At present, there is no information about the role of Atg7 in mammalian RPE autophagy. The fact that inhibiting Atg7 leads to an LLAF increase comparable to that measured after the inhibition of Atg5, implies that both elements participate in a similar way in the regulation of RPE autophagy. The knockdown experiment further confirmed the involvement of autophagy’s effect on lipofuscin and the specific mechanism remains explored.

Studies in recent years have shown that the process of the degradation of ROS by the mammalian RPE cells is rather complex, involving an interaction between phagocytosis and autophagy. Autophagy consists of three distinct biochemical pathways: chaperone-mediated, macroautophagy, and microautophagy [58]. In the present study, by the application of different experiments and methods, we directly investigated and established the importance of the role of macroautophagy in the fate of RPE lipofuscin for the first time and also encountered considerable complexity. Clearly more work is required to understand the subtler mechanistic details. For example, it remains unclear what role the individual components of the mTOR pathway, like mTOR C1 and mTOR C2, play in regulating the autophagic regulation of lipofuscin. Furthermore, the mechanism of interaction between the components of the mTOR pathway and other pathways, such as PIK3 α or Akt/PKB, for lipofuscin degradation remain undetermined. Currently, we are seeking to corroborate the results from the cell culture observation in mouse models of retinal degeneration that accumulate lipofuscin.

The present study has some limitations. For example, in the experiment present in Figure 3, future experiments would be conducted to look at the change in the LC3 pattern after feeding the cells with ROS and then treating them with various stimulators and inhibitors of autophagy, as presented in Figure 1. Using additional markers for the induction of autophagy, like the ubiquitin-binding autophagic adaptor nucleoporin p62, would refine our understanding of the processes involved in the autophagic induction and flux in the RPE cell. Similarly, co-staining with lysosomal markers would lend stronger support to the observation of a punctate appearance after drug treatment, as presented in Figure 3B, currently limited to demonstrating an indication of autophagosome activation by confocal microscopy in a qualitative manner. Another interesting avenue of research would be to pre-treat the cells with stimulators or inhibitors of autophagy and, after feeding with various types of ROS, to monitor the effects of different compounds on autofluorescence buildup. A more powerful suppression of the autophagy makers Atg5 and Atg7 and the additional verification of suppression with RT-PCR, in addition to Western blots, could increase the confidence in the findings related to autophagy inhibition. Another limitation of the current study is that the experiments were done with bovine rhodopsin. Although a recent study has found a close similarity between bovine and human rhodopsin in terms of activation pathways [59], a direct comparison between the autophagy of ROS containing bovine vs. human rhodopsin by ARPE-19 cells has not yet been published, and thus, small differences in the process of autophagy of the two species of rhodopsin cannot be ruled out. It is necessary to consider one more limitation of the current study, related to the results of the live cell imaging presented in this work. Although it is likely that some baseline autofluorescence originates from the lysosomes in living RPE cells, other cellular sources of autofluorescence (e.g., mitochondria) cannot be excluded [60]. In future studies, highly organelle-specific molecular probes may be able to distinguish and quantify the contribution of different cellular compartments to cultured RPE cells not treated with any outer segments.

**Figure 5:**
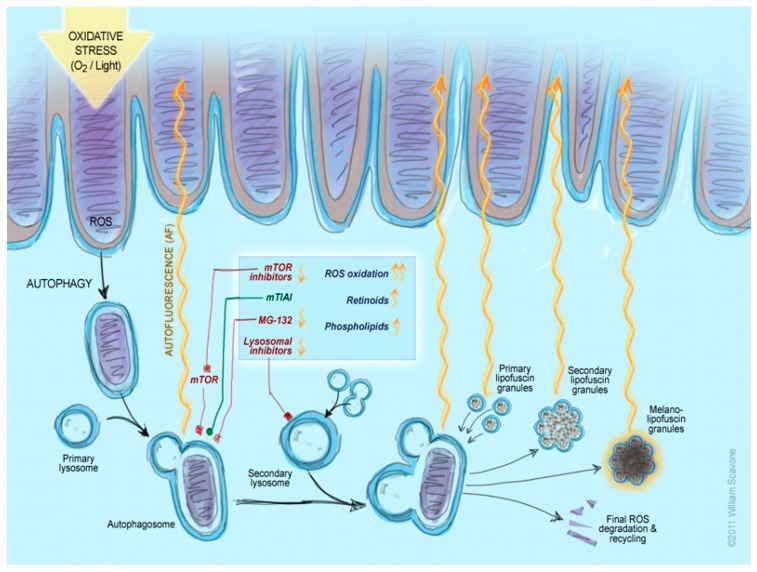
RPE lipofuscin biogenesis and degradation pathways modulated by mTOR activity. Schematic representation of the mTOR modulation on RPE lipofuscin biogenesis and degradation pathways, based on the results from the present study. Initial degradation of the rod outer segment material occurs with the participation of autophagosomes, including their proteosome component. This part of the process can be down-regulated by the mTOR complex or by proteosomal inhibitors, and the application of the latter could increase lipofuscin-like autofluorescence (LLAF). Suppression of mTOR activity (e.g., by application of rapamycin) can stimulate the autophagosome activity and this can lead to a more complete degradation of the material, resulting in decreased LLAF. Similarly, the application of mTOR-independent autophagy inducers can decrease LLAF. In contrast, either direct lysosome inhibition or an oversupply of some ROS components, like phospholipids or retinoids, can lead to increased LLAF. Oxidation of ROS material can also lead to LLAF increase. Abbreviations: Mtch—mitochondrion; Prt—proteasome; mTIAI—mTOR-independent autophagy inducers. The illustration was generated by William Scavone (used with permission).

In summary, based on this work and our recent related studies [26,61], it has been demonstrated that several ROS components contribute to the buildup of lipofuscin-like material in the RPE cell (Figure 5). The formation and degradation of lipofuscin in RPE cells is quite complicated and the regulation of autophagy plays an important role in the degradation. Our data showed a direct pharmacologic influence of mTOR/autophagy pathway on RPE lipofuscin. Furthermore, our data demonstrated that a direct pharmacologic influence on several parts of the mTOR/PIK3alpha/autophagy pathway could lead to a decrease in RPE cell lipofuscin-like autofluorescence. Understanding this pathway and identifying additional drugs to modulate it, can lead to a new therapeutic approach for dry AMD, Stargardt’s disease, and other human lipofuscinopathies.

## 4. Materials and Methods

### 4.1. RPE Cell Culture, ROS Isolation and Modification

ARPE-19 cells were cultured and maintained as previously described [51,52]. Briefly, ARPE-19 cells were procured from the ATCC (Manassas, VA, USA) and grown in high-glucose DMEM (Cellgro/Mediatech Inc., Manassas, VA, USA) supplemented with 10% heat-inactivated fetal calf serum FCS (Sigma-Aldrich, St. Louis, MO, USA) and 1% penicillin/streptomycin (Gibco, Grand Island, NY, USA), at 37 °C in the presence of 5% CO_2_. Cells were routinely subcultured or harvested for experiments using TrypLE Express (Gibco). ROS were prepared from cattle eyes following a described method [26]. The yield was normally 10–20 nmol of rhodopsin per retina, with an OD280/OD500 ratio of 2.3 to 2.6. Oxidized ROS using 4-HNE were prepared as previously described [62]. The protein content of ROS preparations was measured by a BioRad BC kit (Bio-Rad Laboratories, Hercules, CA, USA). The concentrations of protein modifications resulting from this procedure have been previously reported [62]. Modified ROS were stored at −80 °C until use.

### 4.2. RPE Cell Treatment

Post confluent, stationary ARPE-19 cells cultures in 10 cm plates were trypsinized, plated in 24-well or 8-well chamber slides at a confluent density of 1.66 × 10^5^/cm^2^. After an additional culturing for seven days, two different types of ROS were added every day for seven days. The types of ROS were: native ROS or HNE-modified ROS (see above). Unless stated otherwise, the daily dose of ROS was always 4 µg per cm^2^ growth area. The lysosomal inhibitors NH_4_Cl (10 or 20 mM) and chloroquine (20 µM), the proteosomal inhibitor MG-132 (10 µM), the autophagy inhibitor 3-methyladenine (3-MA) (10 mM), and the mTOR pathway inhibitors rapamycin (1 or 10 µM), PI-103 (1 µM), PIK-90 (1 µM), and Ku-0063794 (1 µM) were added separately on two consecutive days after seven days of feeding with ROS. All non-internalized ROS were washed out before the addition of inhibitors/stimulators and this procedure did not interfere with the binding or internalization of ROS.

### 4.3. Flow Cytometry

Flow cytometry was used to evaluate the change in RPE autofluorescence with different treatment. Cells were cultured in 24-well plates and incubated with different components, as described in the section RPE cell culture, ROS isolation and modification. Cells were repeatedly washed, detached with trypsin, and analyzed on a C6 flow cytometer (Accuri Cytometers, Inc. Ann Arbor, MI, USA). A gate was set to exclude cell debris and cell clusters, and 10,000 gated events were recorded. Experiments were performed in triplicate. Two channels were used, with an excitation wavelength of 488 nm: FITC/GFP channel (533/30 nm) and the PE/PI channel (585/40 nm).

### 4.4. RNA Interference

Small interfering RNA (siRNA) oligonucleotides against mTOR, ATG5, and ATG7 were purchased from Cell Signaling Technology (Danvers, MA, USA). For siRNA transfection, cells were seeded at 300,000/well in 24-well tissue dishes, and were transfected with 100 nM of the pooled oligonucleotide mixture by using Lipofectamine (Invitrogen, Carlsbad, CA, USA), following the manufacturer’s protocols. The transfection media were removed after 6 h, and cells were allowed to recover in complete growth media for 36–48 h, before being used in experiments. Western blots were used after three days of transfection, to confirm the effect of RNA interference. Then, the time point at which the decrease in the protein level was most pronounced was determined as three days and, therefore, ROS were supplemented with RPE cells from that time point for another three days, to explore the effects of the suppression of mTOR, ATG5, and ATG7 through RNA interference on RPE cell autofluorescence.

### 4.5. Lentivirus Vectors

Lentiviral vectors were used as a complementary approach to achieve an mRNA inhibition of proteins associated with autophagy. Two lentiviral constructs: shATG5 D6 and shATG5 D9 (Open Biosystems, Huntsville, AL, USA), expressing small hairpin RNAs against Atg5, and two lentiviral constructs: shATG7 84 and shATG7 87 (Sigma-Aldrich), expressing small hairpin RNAs against Atg7, were constructed at the Gene Therapy Center UMASS Medical School. Lentiviral shRNA constructs of mTOR were purchased from Addgene (Cambridge, MA, USA).

pLenti-mCherry-LC3, a construct that produces lentivirus expressing a fusion of the fluorescent protein mCherry (excitation 587 nm; emission 610 nm) and the autophagic marker microtubule-associated protein light chain 3 (LC3) (NM-026160.4), was constructed by cutting out an mCherry-LC3 fragment with SnaBI and EcoRI from pBebe-mCherry-LC3, and then blunting the ends with T4-polymerase, finally ligating into AfeI/XhoI-cut and T4-polymerase-blunted lentivirus construct backbone pLenti. Lentivirus was produced at the Gene Therapy Center of UMASS Medical School following the standard protocol for the transient co-transfection of 293 T cells with the vector plasmid, packaging plasmid, and VSVG envelope plasmid. Briefly, using phosphate calcium methods, the lentivirus vector plasmid (5 μg) and helper plasmids pCMV 8.91 (5 µg)/pCMV-VSVG (3 µg) were co-transfected into 293T cells with 60–70% confluence growing in 100 mm plate. We replaced the culture medium into 7 mL serum-free Opti-MEM one day after transfection, and collected the medium containing lentivirus one day later, then refilled the plate with serum-free Opti-MEM and recollected the medium after another day. The typical lentivirus titer from one 100 mm plate was 105 virus particles/mL ARPE cells in ~40% confluence. Cells were infected with lentivirus for one day by replacing half of the culture medium with lentivirus medium. ARPE cells infected with shRNA lentivirus were seeded at 300,000/well in 24-well tissue dishes. After three days of culture (based on the Western blot’s result), the cells were fed with ROS for three days.

The target sequences of each construct were as follows (5′ to 3′):

shATG5 D6 (TRCN0000151963): GGATGAGATAACTGAAAGG;
shATG5 D9 (TRCN0000151474): GGCATTATCCAATTGGTTT;
shATG7 84 (TRCN0000007584): GCCTGCTGAGGAGCTCTCCAT;
shATG7 87 (TRCN0000007587): CCCAGCTATTGGAACACTGTA;
mTOR1: TTCAGCGTCCCTACCTTCTTCT;
mTOR 2: CCGCATTGTCTCTATCAAGTT.

### 4.6. Analysis of Punctate Cherry-LC3

ARPE-19 cells transfected with the mCherry-LC3 construct were seeded at 1.66 × 10^5^/cm^2^ in an 8-well chamber slide. After three days of cell culture, test drugs (rapamycin, Ku0063794, PI 103, PIK90, NH_4_Cl and chloroquine) were twice added every 24 h and subsequently fixed with 4% PFA, washed three times with 1× PBS, mounted on slides with Vectashield mounting media (Vector Laboratories, Burlingame, CA, USA), and analyzed by confocal microscopy at the UMass Medical School Digital Light Microscopy Core Facility.

### 4.7. Immunoblot Analysis

Cells were lysed in RIPA Lysis Buffer System (Santa Cruz Biotechnology Inc., Santa Cruz, CA, USA). Samples were lysed using five thaw-freeze cycles. Lysates were cleared by centrifugation for 15 min at 4 °C, boiled in SDS sample buffer, resolved using SDS-polyacrylamide gel electrophoresis (S-PAGE), and transferred to the polyvinylidenedifluoride membrane. The membranes were blocked in Odyssey Blocking buffer (LI-COR Biosciences, Lincoln, NE, USA), incubated with the primary antibodies indicated overnight at 4 °C, washed, incubated with conjugated secondary antibodies for one hour, and analyzed by Odyssey Imaging Systems (LI-COR Biosciences). Membranes were blotted with antibodies directed against mTOR, LC3, (all from Cell Signaling) and GAPDH (Millipore, Billerica, MA, USA). Bound antibodies were detected with a goat anti-mouse or rabbit secondary antibody (Odyssey Infrared Image System, LI-COR Biosciences).

### 4.8. Confocal Microscopy

Cells were cultured in 8-well microscopy glass slides (Lab-Tek Chamber Slide; Nunc, Langenselbold, Germany) and treated with different components, as described. After seven days of feeding, cells were repeatedly washed to remove non-internalized ROS, fixed with 4% paraformaldehyde (PFA), stained with 1 mg/mL DAPI (AppliChem, Darmstadt, Germany), and mounted in Vectashield mounting medium (Vector Laboratories, Burlingame, CA, USA). Intracellular lipofuscin granules were documented on a Leica DM 6000 fluorescence microscope (Leica Microsystems, Wetzlar, Germany), using a fluorescein filter set with an excitation of 480/40 nm and an emission of 535/50 nm. Confocal microscopy was performed with a Leica TCS SP5 Spectral point scanning confocal microscope with a Leica DMI 6000 CFS (Confocal Fixed Stage, Leica Microsystems, Wetzlar, Germany). Cells were labeled with 1 µg/mL Hoechst stain (Sigma-Aldrich) for 5–7 min and visualized with the 405, 488, and 561 nm lasers. At the UMass Medical School Digital Light Microscopy Core, confocal microscopy was performed with a Solamere Technology Group (Salt Lake City, UT, USA) CSU10B Spinning Disk Confocal System, which consisted of a CSU10B spinning disk confocal scan head (Yokogawa Electric Corporation, Tokyo, Japan), with high efficiency dichroic mirrors and laser blocking filters attached to a Nikon TE2000-E2 motorized inverted fluorescence microscope equipped with a Nikon’s Perfect Focus System (PFS) (Nikon Instruments, Melville, NY, USA) and a custom acousto-optical tunable filter (AOTF) controlled laser launch, with 405, 488, 561, and 636 nm lasers.

### 4.9. Live Cell Imaging and Fluorescence Quantification

ARPE-19 cells were cultured on 35-mm dishes containing a central 14-mm #1.5 glass coverslip (MatTek, Ashland, MA, USA; P35G-1.5-14-C). Dishes containing cell cultures were transported to the imaging facility and maintained at 37°C by a 20/20 Technology Inc., (Wilmington, NC, USA) objective heater (OHXX) and stage micro-incubator (INC-2000), which also provided a humidified 5% CO_2_–95% air atmosphere. Live cell confocal microscopy was performed on the CSU10B confocal system (see above). Cells were illuminated with an argon ion laser (~4 mWat 488 nm) using a Nikon VC Plan Apo 60× oil objective (NA = 1.4) and the fluorescence emission passed through a 610/60 nm bandpass filter, before entering a RoleraMGi EMCCD 14-bit camera (Qimaging, Surrey, BC, Canada). MetaMorph V7.6.3 (Molecular Devices, Sunnyvale, CA, USA) software was used for equipment control, image acquisition, and image analysis. Cells were imaged before the addition of 50 µL rapamycin (10 µM) or the vehicle (1× PBS), to establish the baseline intensity values and time-lapse imaging continued for 6 h at 30-min intervals. At each time point, a stack of either 16 z-slices (∆z = 0.5 µm) for rapamycin treatment, or 10 z-slices (∆z = 1.0 µm) for the controls (image capture parameters, *t* = 500 ms, EM gain = 4095, gain = 2× for each slice), was acquired. Focus was maintained between time points with Nikon’s PFS, which was turned off during Z-series acquisition.

### 4.10. Image Analysis of Live Cell Imaging

A MetaMorph review of the multidimensional data application was used to convert the Z-series time-lapse data to a maximum intensity projection at each time point. Stacks which contained “hot pixels” were replaced by a local average (Median filter). These pixels did not occur in the areas where the intensity measurements were made. The stack align feature of MetaMorph was used to align subsequent time-lapse images with the first image in the time series, to ensure that measured particles did not move in or out of the measurement regions. The intensity of the aligned stacks was corrected by subtracting the background fluorescence from a non-particle region of each image plane. MetaMorph’s region measurement tool was used to measure the average intensity in four circular regions (diameter = 24.84 µm, area = 480.99 µm^2^).

### 4.11. Statistical Analysis

Al data are presented as means and the error bars indicate the standard deviations. The results from the flow cytometry and the quantification of the results from the Western blots were analyzed using ANOVA and post-hoc Dunnett’s multiple comparisons tests. The analysis was performed using Prism 6 for Windows (GraphPad Software Inc., La Jolla, CA, USA).

## 5. Conclusions

In this study, a seven-day supplementation with native ROS increased lipofuscin-like autofluorescence in cultured RPE cells by more than 30%, while supplementation with HNE-modified ROS increased it by more than 400%, confirming the previous results obtained by our group. Pharmacologic manipulations of autophagy by applying physiological concentrations of autophagy inhibitors/inducers changed LLAF in this system in a predictable way: the inhibition of autophagy increased LLAF, while the induction of autophagy decreased LLAF, and both effects were relatively small (<20%), but statistically significant. These results showed the direct pharmacologic influence of the mTOR/autophagy pathway on RPE lipofuscin and further emphasize the complex role of the different pathways and components involved in autophagy in modulating the process of accumulation and/or degradation of RPE lipofuscin-like material. They also indicate a possibility for the pharmacological manipulation of RPE lipofuscin, which could have important basic science and clinical applications. Understanding this pathway and identifying additional drugs to modulate it, could lead to a new therapeutic approach for dry AMD, Stargardt’s disease, and other human lipofuscinopathies.

## Supplementary Materials

Supplementary materials can be found at www.mdpi.com/1422-0067/18/4/728/s1.
