# Supplementary material for: Inhibition or Stimulation of Autophagy Affects Early Formation of Lipofuscin-Like Autofluorescence in the Retinal Pigment Epithelium Cell
Source: Int J Mol Sci. 2017 Mar 29;18(4):728. doi: 10.3390/ijms18040728 (PMC5412314; doi:10.3390/ijms18040728)
Supplement: Supplementary file 1 [file ijms-18-00728-s001.zip › ijms-169944-suppl.pptx]

## Slide 1
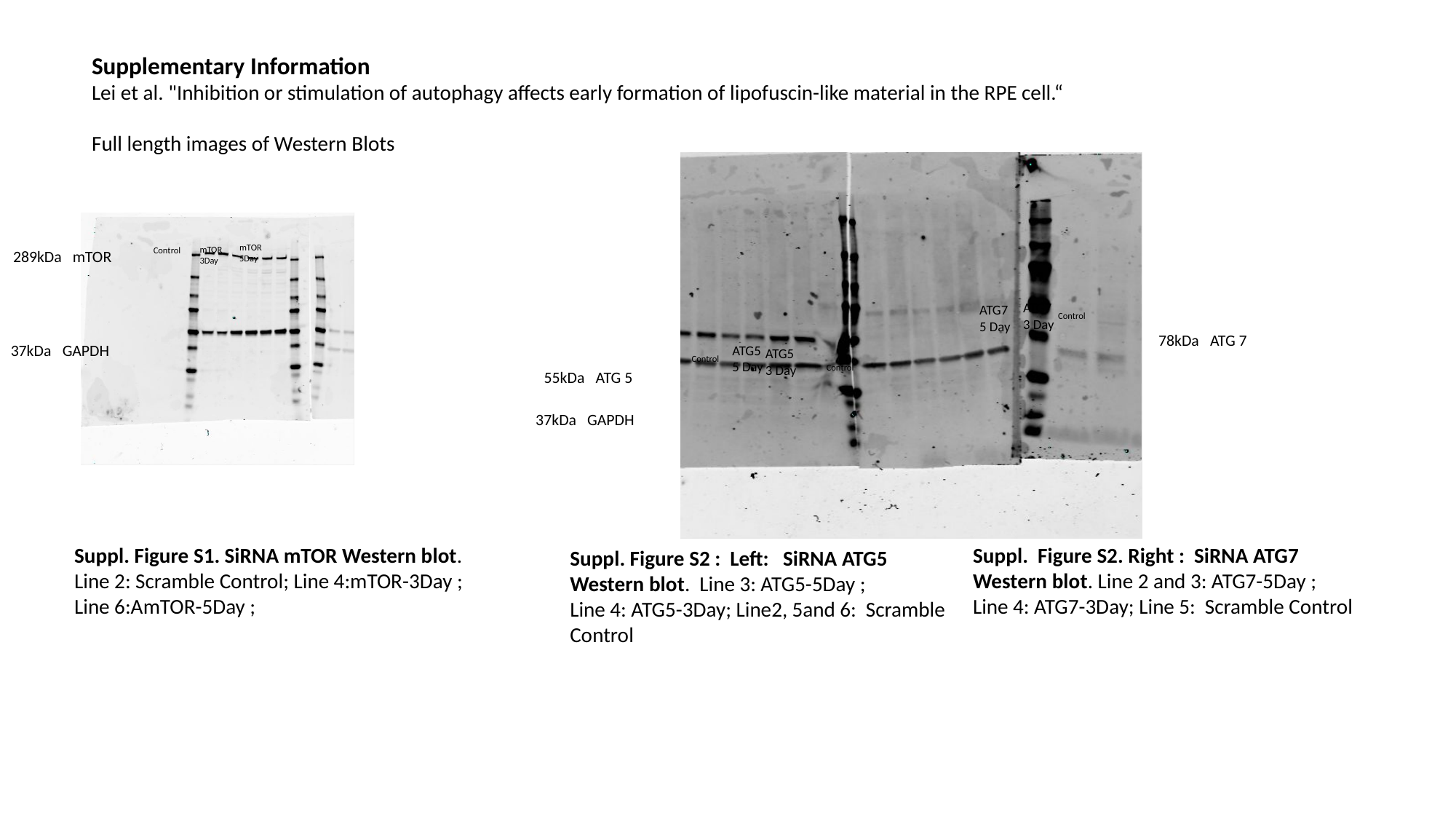

Supplementary Information
Lei et al. "Inhibition or stimulation of autophagy affects early formation of lipofuscin-like material in the RPE cell.“
Full length images of Western Blots
mTOR 5Day
mTOR 3Day
Control
289kDa mTOR
ATG7
3 Day
ATG7
5 Day
Control
78kDa ATG 7
37kDa GAPDH
ATG5
5 Day
ATG5
3 Day
Control
Control
55kDa ATG 5
37kDa GAPDH
Suppl. Figure S1. SiRNA mTOR Western blot. Line 2: Scramble Control; Line 4:mTOR-3Day ;
Line 6:AmTOR-5Day ;
Suppl.  Figure S2. Right : SiRNA ATG7 Western blot. Line 2 and 3: ATG7-5Day ;
Line 4: ATG7-3Day; Line 5: Scramble Control
Suppl. Figure S2 : Left: SiRNA ATG5 Western blot. Line 3: ATG5-5Day ;
Line 4: ATG5-3Day; Line2, 5and 6: Scramble Control

## Slide 2
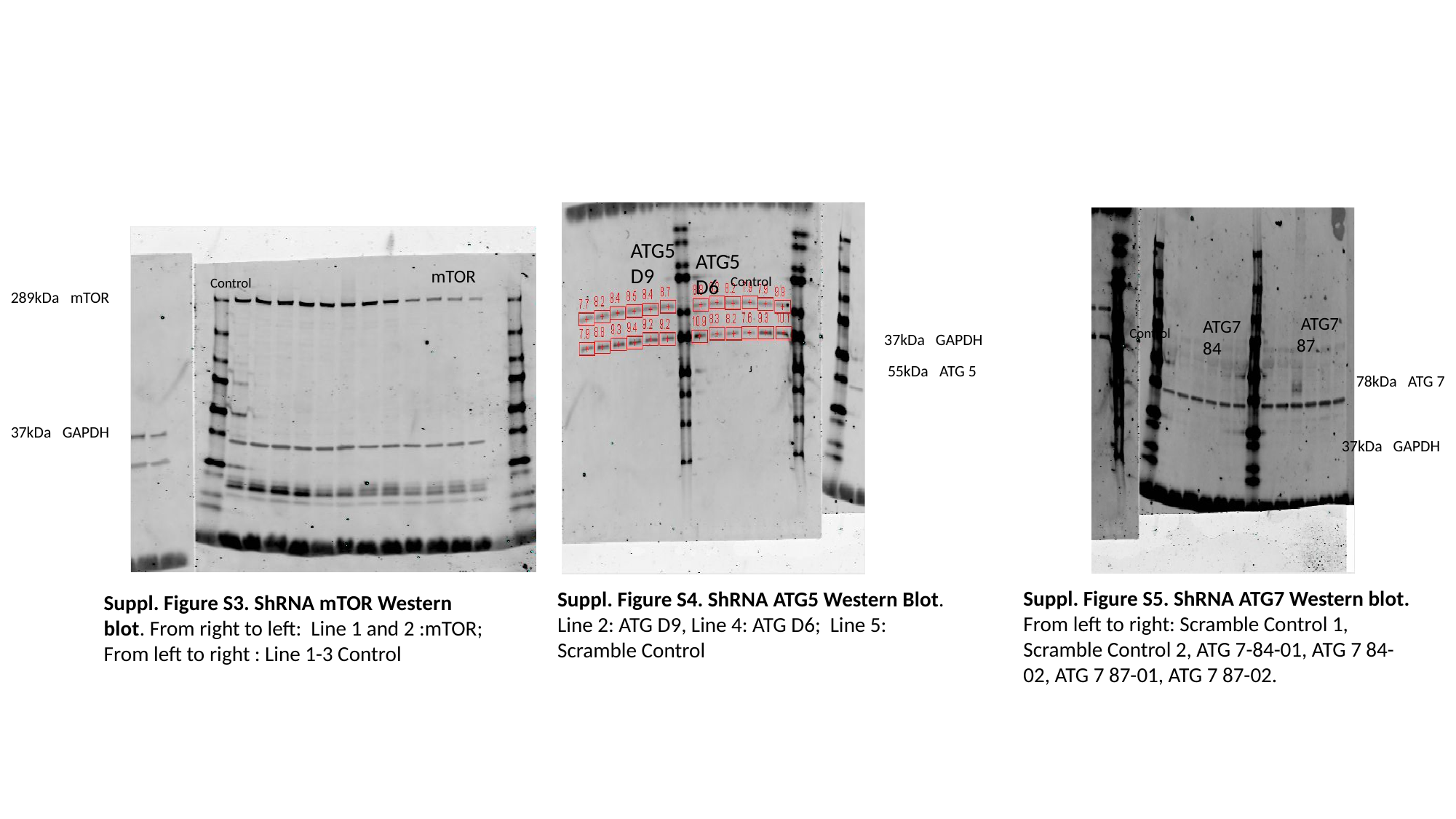

ATG5 D9
ATG5 D6
mTOR
Control
Control
289kDa mTOR
 ATG7 87
 ATG7 84
Control
37kDa GAPDH
55kDa ATG 5
78kDa ATG 7
37kDa GAPDH
37kDa GAPDH
Suppl. Figure S5. ShRNA ATG7 Western blot. From left to right: Scramble Control 1, Scramble Control 2, ATG 7-84-01, ATG 7 84-02, ATG 7 87-01, ATG 7 87-02.
Suppl. Figure S4. ShRNA ATG5 Western Blot. Line 2: ATG D9, Line 4: ATG D6; Line 5: Scramble Control
Suppl. Figure S3. ShRNA mTOR Western blot. From right to left: Line 1 and 2 :mTOR; From left to right : Line 1-3 Control

## Slide 3
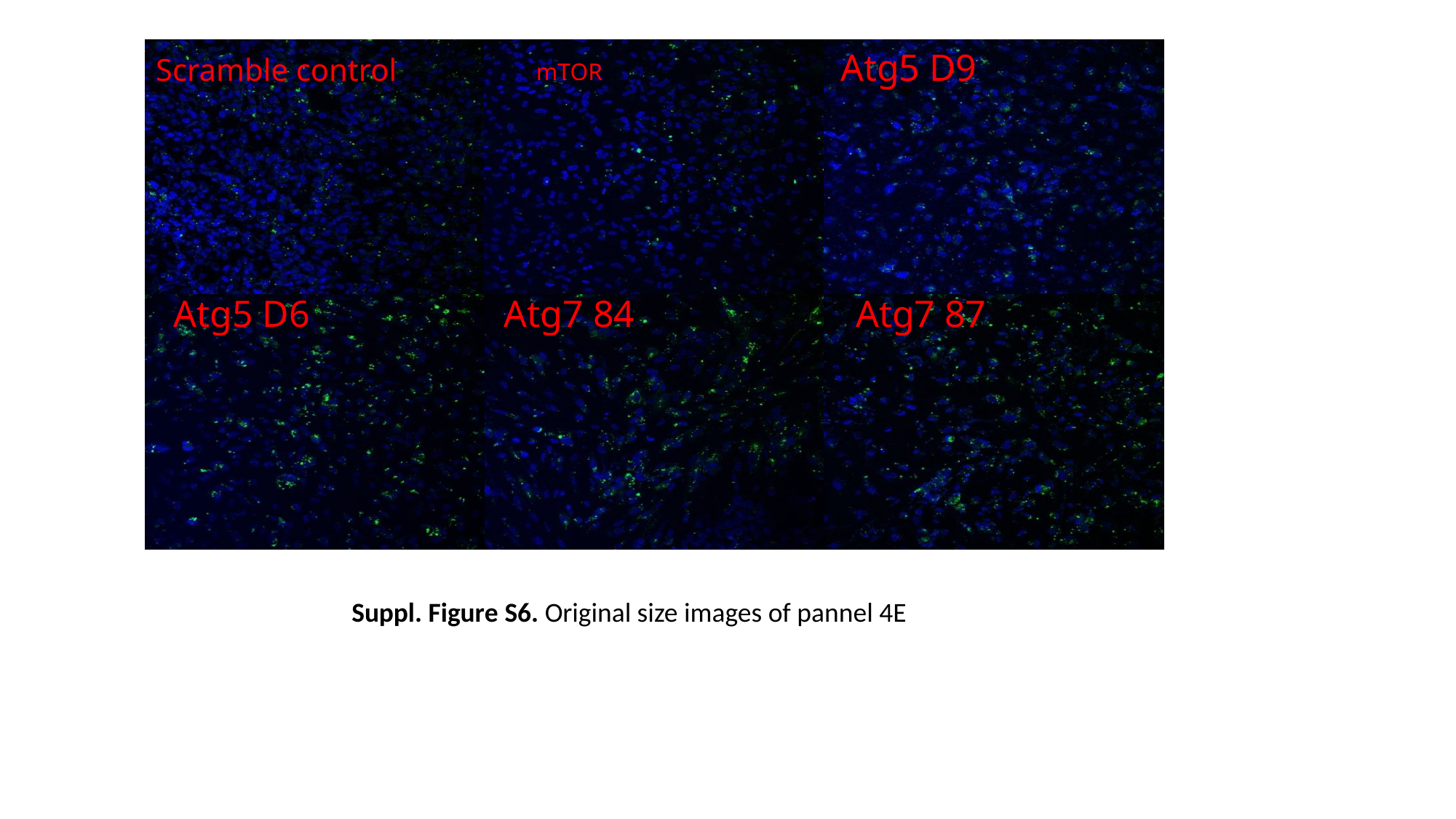

# Scramble control
mTOR
Atg5 D9
Atg5 D6
Atg7 84
Atg7 87
Suppl. Figure S6. Original size images of pannel 4E
