# Supplementary material for: Inhibition or Stimulation of Autophagy Affects Early Formation of Lipofuscin-Like Autofluorescence in the Retinal Pigment Epithelium Cell
Source: Int J Mol Sci. 2017 Mar 29;18(4):728. doi: 10.3390/ijms18040728 (PMC5412314; doi:10.3390/ijms18040728)
Supplement: Supplementary file 1 [file ijms-18-00728-s001.zip › Lei et al_Inhibition or stimulation of autophagy_2016_Suppl Info description.pdf]

Lei et al. Inhibition or stimulation of autophagy affects early formation of lipofuscin-like material in the RPE cell.

Supplementary Information

Supplementary Movie 1. Live cell imaging of ARPE-19 cells (control).
Oblique view of a 3-dimensional representation of the imaged area with the z stacks aligned so that the top surface is closest to the viewer. The time-lapse sequence of image acquisition is presented at 1 frame per second (the acquisition time between frames is ~ 30 min). PBS was added after the first frame. For a 2-dimensional representation of the first and the last frames, see Fig. 2 in the main text.

Supplementary Movie 2. Live cell imaging of ARPE-19 cells treated with Rapamycin.
Same representation as in Supplementary Movie 1. Rapamycin was added after the first frame. For a 2-dimensional representation of the first and the last frames, see Fig. 2 in the main text.
